# Development of ZnO/Na-Montmorillonite Hybrid Nanostructures Used for PVOH/ZnO/Na-Montmorillonite Active Packaging Films Preparation via a Melt-Extrusion Process

**DOI:** 10.3390/nano10061079

**Published:** 2020-05-31

**Authors:** Constantinos Salmas, Aris Giannakas, Petros Katapodis, Areti Leontiou, Dimitrios Moschovas, Andreas Karydis-Messinis

**Affiliations:** 1 Department of Materials Science & Engineering, School of Engineering, University of Ioannina, GR-45110 Ioannina, Greece; dmoschov@cc.uoi.gr (D.M.); karydis.and@gmail.com (A.K.-M.); 2 Department of Food Science and Technology, University of Patras, GR-30100 G. Agrinio, Greece; 3 Department of Biological Applications & Technology, University of Ioannina, GR-45110 Ioannina, Greece; pkatapo@uoi.gr; 4 Department of Business Administration of Food and Agricultural Enterprises, University of Patras, GR-30100 G. Agrinio, Greece; aleontiu@upatras.gr

**Keywords:** active packaging films, food packaging films, zinc oxide nanorods, hybrid nanostructures, nanocomposites, melt-extrusion process, natrium montmorillonite, antimicrobial activity

## Abstract

Nowadays, the shelf-life extension of foods is a topic of major interest because of its environmental and economic benefits. For this purpose, various methods like deep-freezing, ultra-high-temperature pasteurization, drying methods, use of chemicals, controlled-atmosphere preservation, ionizing irradiation, and were investigated. During the last years, the smart packaging for foods using natural biodegradable components is of great interest because it provides positive environmental fingerprint and high shelf-life extension. In the present work, a new nanostructured composite material, the ZnO/Na-Montmorillonite hybrid, was developed. The high antimicrobial properties of the 3-D ZnO material in combination with the high barrier and strength properties of the 2-D Na-Montmorillonite material provided a high promising component for food smart packaging applications. As an extra innovation of this process, the ZnO nanorods coated the external surface of the Na-Montmorillonite and it was not intercalated into the clay as a pillaring material. This new material was incorporated with a 3% *w/w* composition with a biodegradable poly(vinyl)alcohol (PVOH) polymeric matrix which also exhibits antimicrobial activity. The final product was tested via XRD, FTIR, SEM, tensile test, water sorption, water vapor permeability, oxygen permeability UV–vis, and anti-microbial activity tests and it exhibited advanced mechanical and antimicrobial properties, especially for a ZnO/Na-Montmorillonite fraction of 4:1.

## 1. Introduction

Nanotechnology and novel hybrid materials with extraordinary properties are the mainstream trend in food and beverage packaging topics [1]. Thus, antioxidant and antimicrobial agents such as phytochemicals and/or nanoparticles such as ZnO, TiO_2_, and nanoclays are incorporated into active packaging films aiming to enhance tensile properties, increase gas barrier, and gain antimicrobial properties of such packages and thus to extend food shelf-life [2].

Moreover, the green and cyclic economy impose the use of biodegradable materials and natural substances for active packaging applications [3]. Poly(vinyl) alcohol (PVOH) is a synthetic vinylic alcohol polymer which is water-soluble and highly impermeable to gases. This polymer is used as a barrier layer for paper packaging films. During the last years, several studies have been carried out for the development of active packaging films which are based on the incorporation of nanoparticles such as TiO_2_ [4], ZnO [5], clay minerals essential [6] oils [7], and phytochemicals [8] on PVOH matrix.

Nanoclays are the most commonly used 2-D nanomaterials for active food packaging applications. The global nanoclay market for food packaging was the largest segment in 2014, accounting for USD 343.0 million, and is expected to grow significantly during 2022 [9]. Incorporation of nanoclays into a polymer matrix leads to polymer/biopolymer nanocomposites with enhanced tensile and barrier properties [2].

Compared to the inorganic 0-D nanomaterials such as nanoparticles of Ag, Cu_2_O, CuO, TiO_2_, and MgO, which are exhibiting antibacterial properties, the zinc oxide (ZnO) is the most commonly used compound for active food packaging applications [10]. ZnO is one of the five zinc compounds which are registered by the US FDA (Food and Drug Administration) in the GRAS (Generally Recognized As Safe) list (FDA, 2011).

In this study, the development of a new fully biodegradable and low-cost material for active packaging applications is presented. The main innovation of this work was the coating of the external surface of commercial sodium montmorillonite (NaMt) with ZnO nanorods which exhibit antimicrobial activity. The NaMt material is one of the most usually used materials as a nano-reinforcement and barrier agent. Thus, the final target of this work was to obtain a hybrid nanostructure with increased mechanical strength and barrier properties due to the presence of the nanoclay and with increased antimicrobial properties due to the presence of the zinc oxide nanorods on the surface of the nanoclay. Five batches of ZnO/NaMt hybrid nanostructures, each one with different ZnO/NaMt nominal wt % ratio, were developed. Sequentially, the obtained ZnO/NaMt hybrid nanostructures were incorporated into the PVOH biodegradable polymeric matrix via a melt-extrusion process aimed at the development of an advanced active food packaging film. The success of this attempt was evaluated by the following steps: (1) The characterization of the obtained ZnO/NaMt hybrid nanostructures with XRD, FTIR, and SEM methods to figure out the mechanism of growth of the ZnO nanorods on NaMt surface. (2) The characterization of the developed PVOH/ZnO/NaMt nanocomposite films with XRD, FTIR spectroscopy. This step aims to investigate of the distribution of the ZnO/NaMt nanohybrids in the PVOH matrix. (3) The evaluation of tensile properties, water/oxygen barrier properties, and antimicrobial activity of the developed PVOH/ZnO/NaMt nanocomposite films. This step indicated to us the optimum ZnO/NaMt nominal wt % ratio for the development of the most promising packaging film with advanced mechanical, water barrier, and antimicrobial properties.

## 2. Materials and Methods

### 2.1. Materials

Poly(vinyl alcohol) (PVOH) with low molecular weight (13.000−23.000) and hydrolysis degree of 87−89%, zinc acetate dihydrate (Zn(CH_3_COO)_2_×2H_2_O) and Ammonia solution 25% were purchased from SIGMA-ALDRICH, Co., 3050 Spruce Street, St. Louis, MO 63103 USA 314-771-5765. Sodium exchanged montmorillonite (NaMt) with the code name Nanomer^®^ PGV with mass density 2.6 g/cm^3^ and CEC value of 145 meq/100 g produced by Nanocor Inc. 2870 Forbs Avenue Hoffman Estates, IL 60192 United States and supplied by Sigma-Aldrich. The chemical composition of NaMt was 62.9% SiO_2_, 19.6% Al_2_O_3_, 3.35% Fe_2_O_3_, 3.05% MgO, 1.68% CaO, and 1.53% Na_2_O.

### 2.2. Preparation

#### 2.2.1. Preparation of ZnO/NaMt Hybrid Nanostructures

The method which was followed for the growth of ZnO nanorods was based on a previous report [11]. Five different ZnO/NaMt weight ratios were prepared (Table 1). In all cases, 4.525 g of Zn(CH_3_COO)_2_×2H_2_O (24.7 mmol) was used to obtain approximately 2 g of ZnO nanorods. The amount of the NaMt used was 0.0, 0.5, 1.0, 2.0, 3.0, 4.0, and 5.0 g (see Table 1) to obtain final ZnO/NaMt nanohybrids with low, medium, and high NaMt loadings. An amount of 4.525 g of Zn(CH_3_COO)_2_×2H_2_O (24.7 mmol) was dissolved in 50 mL deionized water in a spherical glass flask and it was stirred for 5 min. A second aqueous solution of NH_3_ 25% *w/w* was slowly added to the first in a drop-wise manner, stirring constantly, to keep the pH at ~11. A white precipitate was initially observed, but sequentially it was dissolved back into the solution because of the addition of the NH_3_ (approx. 11 mL). Then, an appropriate amount of NaMt was spread into the clear solution which was further stirred for 2 h. The obtained slurries were refluxed and aged for 1 h and the ZnO/NaMt precipitates were washed several times with deionized water to remove ammonia excess and dried at 60 °C for 24 h. All the used amounts of Zn(CH3COO)_2_×2H_2_O and NaMt, the typical conditions for the preparation, and the code names of ZnO/NaMt nanohybrids are listed in Table 1.

#### 2.2.2. Preparation of PVOH/ZnO/NaMt Nanocomposite Films

PVOH/ZnO/NaMt blends were prepared in a lab-scale, twin-screw extruder (Haake Mini Lab II, ThermoScientific, ANTISEL, S.A., Athens, Greece). The ZnO/NaMt content was fixed at 3% *w/w*. The blending process was carried out at a temperature of 190 °C, for 10 min blending time and a rotor speed of 100 rpm. In Table 2, we can find the adopted code names, the used amounts of PVOH and ZnO/NaMt nanohybrids, the % nominal content of ZnO and NaMt in the obtained films and finally the extrusion process conditions for the preparation of all composites in this work. The blends, which were produced from the lab-scale twin-screw extruder, were hot-pressed into films for 5 min at 185 °C under 2 MPa constant pressure using a hydraulic press with heated plates.

### 2.3. XRD Analysis

All the produced ZnO/NaMt nanostructures, as well as the developed PVOH/ZnO/NaMt ternary films, were characterized by the XRD pattern obtained using a Brüker D8 Advance X-ray diffractometer (Bruker, Analytical Instruments, S.A., Athens, Greece) which was equipped with a LINXEYE XE High-Resolution Energy-Dispersive detector.

### 2.4. FTIR Spectrometry

The chemical structure of ZnO/NaMt nanostructures, as well as the developed PVOH/ZnO/NaMt ternary films, were confirmed by IR spectra measurements. An FT/IR-6000 JASCO Fourier transform spectrometer (JASCO, Interlab, S.A., Athens, Greece) was employed and worked in the frequency range of 4000–400 cm^−1^. The measured infrared (FTIR) spectra were the average of 32 scans at 2 cm^−1^ resolution.

### 2.5. SEM Images

The surface morphology of the obtained ZnO/NaMt nanostructures, as well as the average length of the ZnO nanorods, were obtained using a JEOL JSM-6510 LV SEM Microscope (Ltd., Tokyo, Japan) equipped with an X-Act EDS-detector by Oxford Instruments, Abingdon, Oxfordshire, UK (an acceleration voltage of 20 kV was applied). Before SEM observation, the materials were dissolved in EtOH, sonicated for 30 min (to avoid aggregation), and dropcasted on silicon substrates.

### 2.6. Tensile Properties

Tensile measurements were carried out for all prepared PVOH/ZnO/NaMt films using a Simantzü AX-G 5kNt instrument (Simandzu. Asteriadis, S.A., Athens, Greece) and according to the ASTM D638 method. Three to five samples of each one film were tensioned at an across head speed of 2 mm/min. The sample shape was a dumb-bell with gauge dimensions of 10 × 3 × 0.22 mm. Force (N) and deformation (mm) were recorded during the test, and the stress, stain, and modulus of elasticity values were calculated based on these measurements and the gauge dimensions.

### 2.7. Water Sorption

Selected films were cut into small pieces (20 × 20 mm), desiccated overnight under vacuum, and weighed to determine their dry mass. The weighed films were placed in closed beakers containing 50 mL of deionized water and stored at T = 25 °C. The total water sorption value was calculated by a periodical weighting of the samples until the saturation with water was reached. The calculations were carried out according to the equation:

(1) $$W.G.\left(\%\right)=\frac{mWet-mDry}{mDry}\times 100$$

where *mWet* and *mDry* are the weight of the wet and dry film, respectively, and *W.G.* is the Water Gain.

### 2.8. Water Vapor Permeability (WVTR)

Water vapor permeability of all PVOH/ZnO/NaMt films was determined at 38 °C and 50% RH according to the ASTM E96/E 96M-05 method using a handmade apparatus and following the methodology described extensively in our previous publications [12,13,14,15,16,17,18]. Each film with approx. 2.5 cm in diameter and approx. 200 µm average thickness was placed on the top of a one-open end cylindrical tube made of plexiglass which contained dried silica gel inside and was sealed by a rubber O-ring. The test tube was placed in a glass desiccator with a 200 mL saturated magnesium nitrate solution (50% relative humidity (RH)). Test tubes were weighed periodically for 24 h and the WVTR was calculated according to the following equation:

(2) $$WVTR=\frac{G/t}{A}$$

where: *G* is the weight increase of the tested tubes in grammars, *t* is the time in hours, *G/t* is the slope of the linear function ΔG = f(t), and A is the permeation area of the film. Additionally, the weight of the tested films was measured before and after the WVTR test to exclude any absorption phenomena of humidity by the film.

### 2.9. Oxygen Permeability

The oxygen transition rate (OTR) was measured using an oxygen permeation analyzer (8001, Systech Illinois Instruments Co., Johnsburg, IL, USA). The examined samples were tested at 23 °C and 0% RH according to the ASTM D 3985 method. OTR values were measured in cc O_2_/m^2^/d. The oxygen permeability (OP) values of the tested samples were calculated by multiplying the OTR values with the average film thickness, which was approximately 300–400 μm. The OTR value for each kind of film resulted from the mean value of measurements of three pieces.

### 2.10. UV–Vis Absorbance Analysis of Films

UV–vis absorbance measurements were carried out for all the PVOH/ZnO/NaMt nanocomposite films as well as for the pure PVOH and PVOH/NaMt nanocomposite films using a Shimatzu 1900 spectrophotometer. The range of the absorbance wavelength was from 200 to 800 nm. During the analysis, the scan rate and the spectral bandwidth were fixed to be 50 nm/min and 2 nm, respectively. The dimension of the samples which were used for this analysis was 50 mm length and15 mm width.

### 2.11. Antimicrobial Activity Tests

Escherichia coli strain BL21(DE3) was taken from a culture collection of the Department of Biological Applications and Technologies, University of Ioannina. The bacterial strain was recovered from cryo-preservation, was grown on Luria–Bertani (LB) agar at 37 °C, and stored on LB agar slants at 4 °C. Luria–Bertani (LB) was purchased from Lennox (LAB173) and Luria–Bertani agar was purchased from LAB which are NEOGEN companies both of them (NEOGEN Co. 620 Lesher Place, Lansing, MI 48912 USA, 800.234.5333 USA).

The antibacterial activity of PVOH/ZnO/NaMt as well as of pure PVOH and PVOH/NaMt nanocomposite films against *E. coli* was tested according to the method of Karageorgou et al. with some modifications [19]. Approximately, 5 mL of 0.9% NaCl solution containing 10^7^ CFU/mL bacterial cells at the exponential phase were deposed on each film (33 mm diameter) in a glass petri dish (60 × 15 mm) to interact for 12 h at 25 °C, under shaking at 100 rpm. As a control, we treated bacteria samples in the same conditions without the presence of any film. After the treatment, 25 μL of each sample was added at Nunclon™ Delta 96-Well MicroWell™ Plates from Thermo Scientific, ANTISEL, S.A., Athens, Greece, containing 225 μL sterile LB broth medium and the growth curves at 37 °C were determined based on the absorbing value of OD_600_. All experiments were performed in triplicate wells for each film and repeated at least twice. The lethal effect of each film is defined as the percentage growth inhibition of treated cells compared to control at the exponential growth phase.

## 3. Results

### 3.1. Characterization of ZnO/NaMt Hybrid Nanostructures

**Figure 1:**
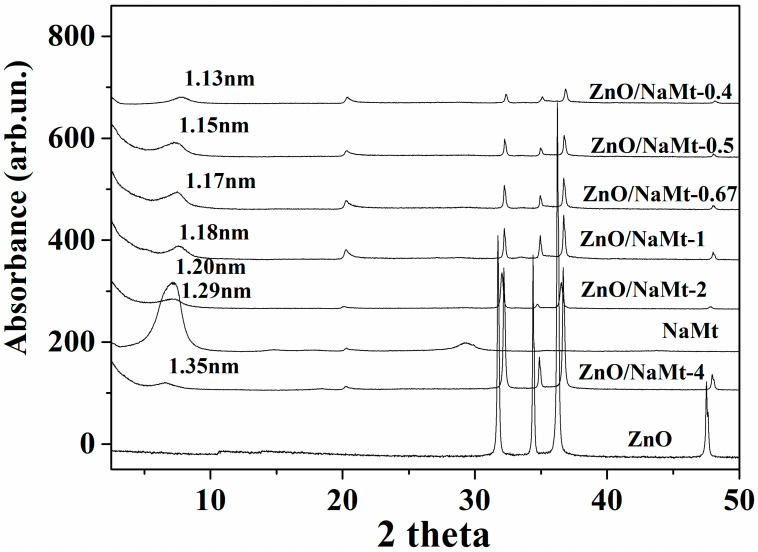
Comparison of XRD plots of all the obtained ZnO/NaMt nanohybrids with the XRD plot of the pure NaMt and with the XRD plot of the pure ZnO material. The 2theta range is between 2° and 50°.

Figure 1 shows the X-ray diffraction patterns of ZnO, NaMt, and ZnO/NaMt nanohybrids in a 2theta angle range from 2theta=2° to 2theta=50°. The XRD peaks at 2theta angles around 31.7°, 34.4°, 36.2°, and 47.5°, correspond to the (100), (002), (101), and (102) reflections of Hexagonal: P63mc Zinc Oxide wurtzite crystal phase (COD-2015 library, Crystallography Open 139 Database). The (002) reflections originate from the vertically oriented ZnO nanowires while the (101) reflections originate from the tilted nanowires. The increasing of the (002) peak indicates well-oriented ZnO nanowires [20,21]. These peaks, which were recorded in all XRD plots of the ZnO/NaMt nanohybrids, indicated the growth of ZnO nanorods on NaMt. Additionally, in all XRD plots of ZnO/NaMt nanohybrids, the basal spacing (d_001_) of NaMt was observed at a low angle region around 7.3°. The zero increase of the basal space of all ZnO/NaMt nanostructures as compared to the basal space of the pure NaMt indicates that no change was taking place in interlayer NaMt space after the ZnO growth. The calculated values of basal space are shown in Figure 1. This result is in contrast with previous reports where the preparation of ZnO/NaMt via zinc chloride solution led to the intercalation of ZnO nanoparticles into the NaMt galleries [22]. This fact indicates that in our case the ZnO nanorods do not intercalate in the NaMt galleries and coated the external surface of the NaMt platelets. Apart from the XRD measurements and of the SEM images, this result is supported by the findings reported elsewhere [23] where the Zn(CH_3_COO)_2_x4H_2_O was used as starting material for ZnO growth as in our case and the ZnO nanorods coated the external surface of the halloysite nanoclay. Moreover, XRD plots of all ZnO/NaMt nanohybrids exhibit a peak-shift to higher angles for ZnO characteristic peaks. This phenomenon is common when you dope a material because impurities are introduced in the crystal lattice. Additionally, it is common after the processing of materials because of residual tensile stresses presented in thin layers [24,25,26]. According to previous reports [22,27,28] for all ZnO/NaMt nanohybrids, an increase to NaMt content causes a decrease in the characteristic crystal phase peaks of ZnO. This result indicates that the higher the content of NaMt the higher the covering of ZnO crystals.

**Figure 2:**
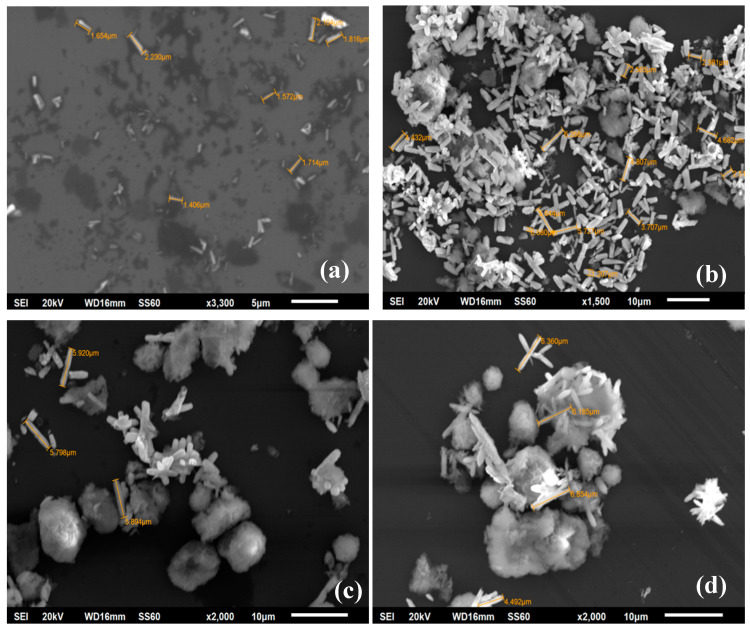
SEM images for ZnO/NaMt nanohybrids with different compositions which are specified in Table 2. White sticks are the ZnO nanorods, bulk light-gray mass is the NaMt clay and the dark-gray ground is silica wafer used as a substrate for SEM measurements. (**a**) ZnO/NaMt-4, (**b**) ZnO/NaMt-2, (**c**) ZnO/NaMt-0.5, and (**d**) ZnO/NaMt-0.4.

Representative SEM images of ZnO/NaMt-4, ZnO/NaMt-2, ZnO/NaMt-0.5, and ZnO/NaMt-0.4 hybrid nanostructures are illustrated in Figure 2. As it is obvious from SEM images, by increasing NaMt content, the obtained crystal size of ZnO nanorods is increased. For ZnO/NaMt-4 the obtained length of ZnO nanorods range from 1.5 to 2.1 μm, for ZnO/NaMt-2 from 2.3 to 4.0 μm, for ZnO/NaMt-0.5 from 5.7 to 5.9 μm, and ZnO/NaMts-0.4 from 6.3 to 6.8 μm. SEM images have also shown that the ZnO nanorods coated the external surface area of the NaMt platelets and with different orientations. This result is consistent with the XRD findings discussed above. The preparation method which was followed led to ZnO/NaMt nanohybrids with free NaMt’s basal space. This space was available to encapsulate biopolymer molecules.

**Figure 3:**
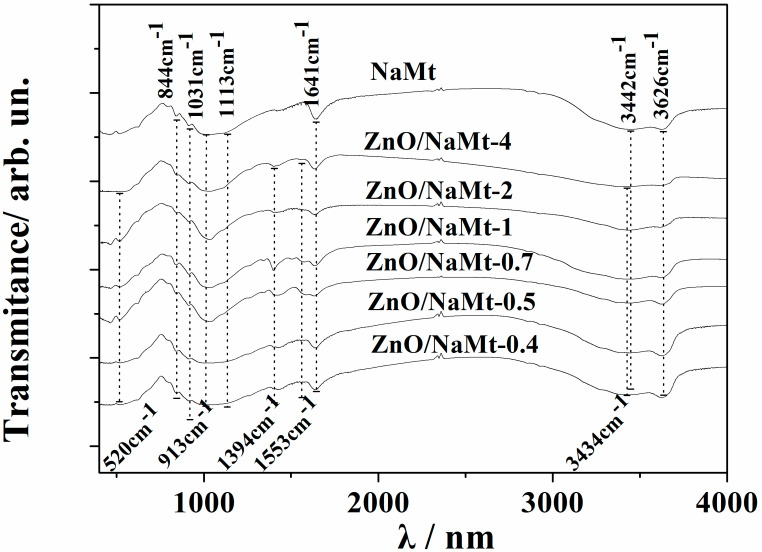
Comparison of FTIR plots of all obtained ZnO/NaMt nanohybrids with the FTIR plot of the pure NaMt.

Figure 3 shows the FTIR spectra of all ZnO/NaMt nanohybrids as well as the FTIR spectra of pure NaMt. The characteristic absorption band of NaMt is assigned, at ~3626 cm^−1^ to OH group stretching bonded with Al^3+^ cation [29]; at ~3442 cm^−1^ to the H_2_O stretching vibrations; at ~1641 cm^−1^ to the H_2_O bending vibrations; and at ~1113 cm^−1^ and ~1031 cm^−1^ to the SiO stretching vibrations [30]. Moreover, the three bands at 913, 879, and 844 cm^−1^ are OH bending modes, the band at ~913 cm^−1^ is the bending mode of AlAl–OH; at ~879 cm^−1^ is the bending mode of AlFe–OH, and finally at ~844 cm^−1^ is the bending mode of FeFe–OH [30,31].

All the obtained spectra of ZnO/NaMt nanohybrids exhibit an absorption band at around 520 cm^−1^, which is the typical characteristic band of pure wurtzite hexagonal phase ZnO [32,33] and an absorption band at 3434 cm^−1^, which corresponds to the O–H mode [6]. The two strong peaks at 1553 cm^−1^ and 1394 cm^−1^ are assigned to the symmetric starching of the carboxylate group (COO^−^) which originates probably from a small residue of zinc acetate that was used for the growth reaction [33]. The characteristic absorption band of OH group stretching bonded with Al^3+^ cation at ~3626 cm^−1^ decreases as the ZnO content increases (see Figure 3). This fact suggests that the coating of the NaMt surface with ZnO nanorods probably covers the Al–OH surface group leading to a decrease of the corresponding stretching band of the NaMt.

### 3.2. Characterization of PVOH/ZnO/NaMt Nanocomposite Films

**Figure 4:**
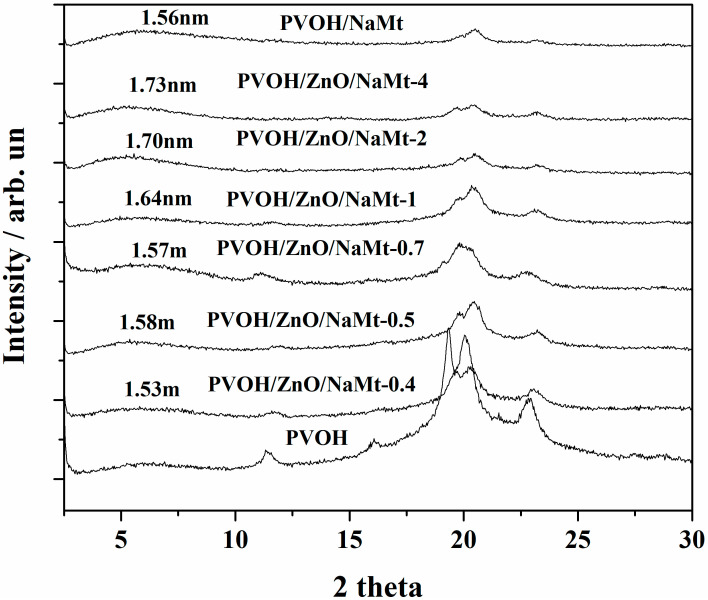
Comparison of XRD plots of all PVOH/ZnO/NaMt nanocomposite films with the XRD plot of the PVOH/NaMt film and with the XRD plot of the pure PVOH film.

Figure 4 shows the XRD plots of all obtained PVOH/ZnO/NaMt nanocomposite films as well as of “blank” PVOH/NaMt samples and pure PVOH film (gray line). All PVOH/ZnO/NaMt nanocomposite films as well as the “blank” PVOH/NaMt sample exhibit a shift of the NaMt characteristic peak at 2theta around 5°–6° and a PVOH characteristic peak attenuation. This fact indicates the incorporation of PVOH chains into the free interlayer space of ZnO/NaMt nanohybrids and the formation of an intercalated nanocomposite structure. PVOH/ZnO/NaMt-4 and PVOH/ZnO/NaMt-2 samples were the nanocomposite films with the lowest NaMt content. These samples showed the highest increase of NaMt d-spacing which was 1.73 nm and 1.70 nm, respectively. As the NaMt content in PVOH/ZnO/NaMt films increased, the d-spacing decreased, indicating partial destruction of obtained intercalated nanocomposite structure.

**Figure 5:**
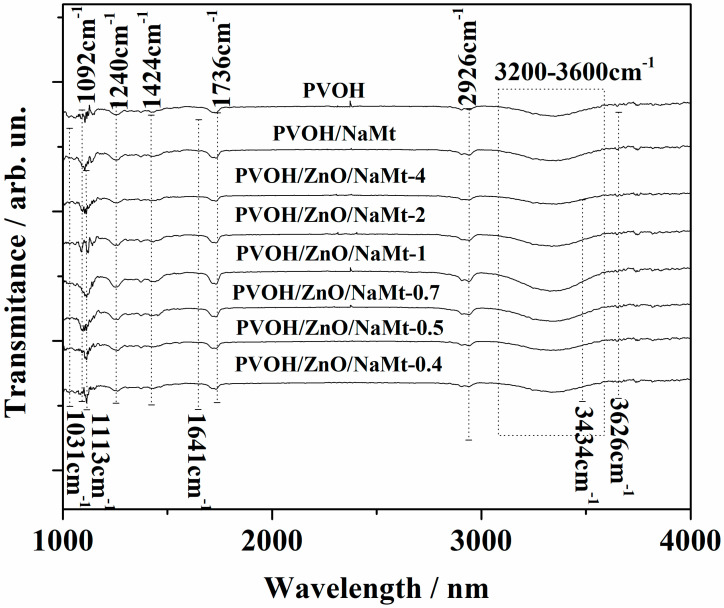
Comparison of FTIR plots of all obtained PVOH/ZnO/NaMt nanocomposites films with the FTIR plot of the PVOH/NaMt nanocomposite film and with the FTIR plot of the pure PVOH film.

Figure 5 Represents the FTIR spectra of pure PVOH and PVOH/ZnO/NaMt nanocomposite films. The characteristic bands of all the FTIR plots of the PVOH’s are as follows: from 1020 to 1092 cm^−1^ the C−OH stretching vibration, at 1240 cm^−1^ the C–H wagging vibration, at 1424 cm^−1^ the O−H in-plane bending vibration, at 1736 cm^−1^ the Carbonyl (C=O) stretching vibration, at 2926 cm^−1^ the C−H alkyl stretching vibration, and from 3000 to 3600 cm^−1^ the strong broad absorption band of O−H stretching vibration. These results are in agreement with others in previous reports [34,35,36]. The presence of NaMt in PVOH/ZnO/NaMt films is indicated by the appearance of the characteristic absorption bands at ~3626 cm^−1^, at ~1641 cm^−1^, and at ~1031 cm^−1^. Because these absorption bands are very low, they are more obvious on PVOH/NaMt, PVOH/ZnO/NaMt-0.5, and PVOH/ZnO/NaMt-0.4 samples where the wt % content of NaMt is the highest. The presence of ZnO nanorods is indicated by the broadening of PVOH’s absorption band at 3000–3600 cm^-1^ which is caused by the absorption band of ZnO’s O–H mode at 3434 cm^−1^. This broadening is more obvious in PVOH/ZnO/NaMt-5 and PVOH/ZnO/NaMt-4 samples where the ZnO wt % content is the highest. The main difference between the FTIR plots of all PVOH/ZnO/NaMt and PVOH/NaMt nanocomposites and the FTIR plot of pure PVOH is the broadening of the PVOH’s hydroxyl groups vibration band which is located at 3000–3600 cm^-1^. This broadening indicates the interaction of PVOH chains with the inside galleries surface of the NaMt clay platelets. 

**Figure 6:**
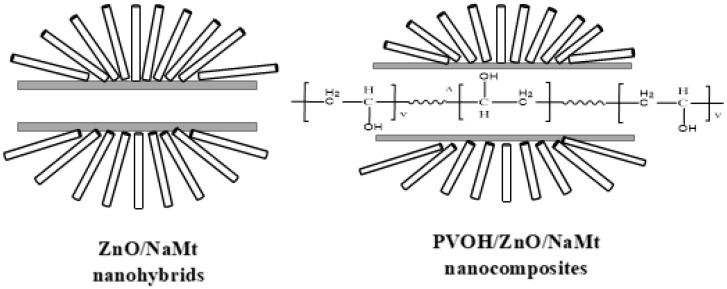
Schematic representation of ZnO nanorods growing on the external surface of the NaMt clay (**left**) and for the incorporation of the PVOH chains in the free interlayer gallery space of the ZnO/NaMt nanohybrids (**right**).

The major conclusion from the characterization measurements which were carried out on both ZnO/NaMt nanohybrids and PVOH/ZnO/NaMt nanocomposites was that ZnO nanorods which coated the external surface area of NaMt platelets tilted to different orientations. Moreover, the intercalation of NaMt interlayer space with ZnO nanorods was practically zero. This free interlayer space of ZnO/NaMt nanohybrids was occupied from PVOH chains and a new intercalated nanocomposite structure was developed. This scenario is graphically illustrated in Figure 6.

### 3.3. Tensile Properties of PVOH/ZnO/NaMt Nanocomposite Films

**Figure 7:**
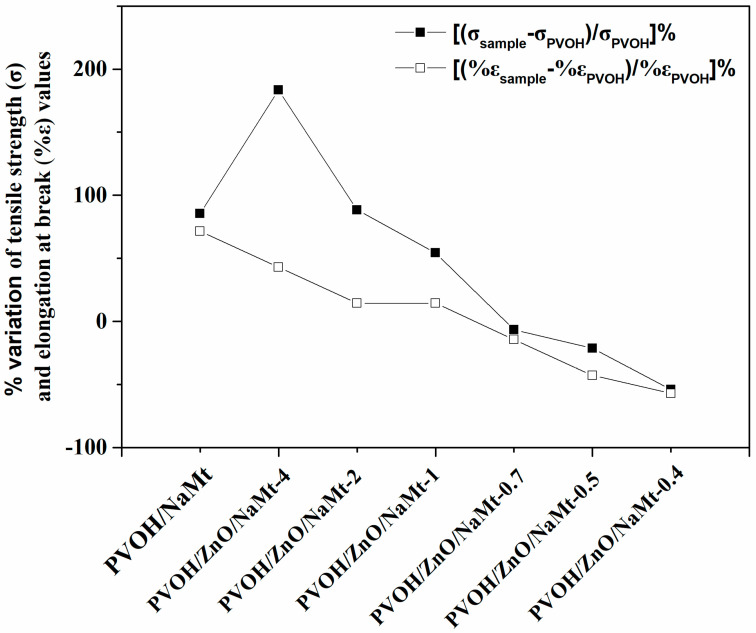
Comparison of the variation of % tensile strength (σ) and % elongation at break (%ε) values of all the obtained PVOH-based nanocomposite films from the respective values of the pure PVOH film which was used as reference material.

In Table 3, the calculated modulus of elasticity (E), the tensile strength (σuts), and the % elongation at break (%ε), of all tested PVOH/ZnO/NaMt nanocomposite films as well as of PVOH/NaMt nanocomposite and pure PVOH film are presented. A comparison of the calculated values of % variation of modulus of elasticity (E), tensile strength (σuts), and % elongation at break (%ε) between nanocomposite films and pure PVOH film is depicted ιn Figure 7. As it is obvious from this figure, the tensile strength of PVOH/NaMt, PVOH/ZnO/NaMt-4, PVOH/ZnO/NaMt-2, and PVOH/ZnO/NaMt-1 samples increased up to 85.5%, 183.4%, 88.4%, and 54.3%, respectively. The same parameter decreased up to −6.5%, −21.4%, and −54.0% for PVOH/ZnO/NaMt-0.7, PVOH/ZnO/NaMt-0.5 and PVOH/ZnO/NaMt-0.4 samples, respectively. A similar trend is observed for the % variation of % elongation at break parameter. Thus, the increase of wt % of ZnO content led to enhanced tensile strength and elongation at break properties of PVOH/ZnO/NaMt nanocomposites. Additionally, the increase of wt %. NaMt content led to increased brittleness of PVOH/ZnO/NaMt nanocomposites. The enhancement of both tensile strength and elongation at break values by the increase of the wt %, ZnO content in PVOH/ZnO nanocomposites is in agreement with results reported in other papers [37,38]. Additionally, the enhancement of tensile strength and the decrease of elongation at break values by the increase of the % NaMt content in the PVOH matrix consists of results reported previously [36].

The tensile strength values of PVOH/ZnO/NaMt-4, PVOH/ZnO/NaMt-2, and PVOH/ZnO/NaMt-1 nanocomposite increase compared to the tensile strength value of pure PVOH. Additionally, as it is mentioned in the previous paragraph, the XRD results indicated an intercalated nanocomposite structure for PVOH/ZnO/NaMt-4, PVOH/ZnO/NaMt-2, and PVOH/ZnO/NaMt-1 nanocomposites. This result indicates enhanced tensile strength properties, according to the XRD theory. Moreover, the highest strengthening was observed for PVOH/ZnO/NaMt-4 and PVOH/ZnO/NaMt-2 samples. For these two films, XRD results indicated the highest d-spacing values of NaMt, (see Figure 4). The decreased tensile strength values of PVOH/ZnO/NaMt-0.7, PVOH/ZnO/NaMt-0.5, and PVOH/ZnO/NaMt-0.4 samples as compared to the tensile strength of the pure PVOH sample is consistent with the partial destruction of the intercalated nanocomposite structure which was observed for these samples via XRD measurements. The similarity of the trend of d-spacing values with the trend of tensile strength values of all PVOH/ZnO/NaMt nanocomposite films is a confirmation for the validity of the results of this study.

### 3.4. Water Sorption

Table 4 presents the % water sorption values of all films after immersion in water. As it is obvious, the pure PVOH film and the PVOH/NaMt nanocomposite film absorbed huge amounts of water and were almost damaged after 2−3 h of immersion in water. Lower % water sorption values were obtained for PVOH/ZnO/NaMt nanocomposite films. The % water sorption values of PVOH/ZnO/NaMt films decreased as the % content of ZnO increased. The lowest % water sorption value is obtained for the PVOH/ZnO/NaMt-4 sample which had the highest % ZnO nanorods content. The surface water resistance properties of ZnO nanorods are well known in the literature [39].

### 3.5. Water and Oxygen Barrier Properties

**Figure 8:**
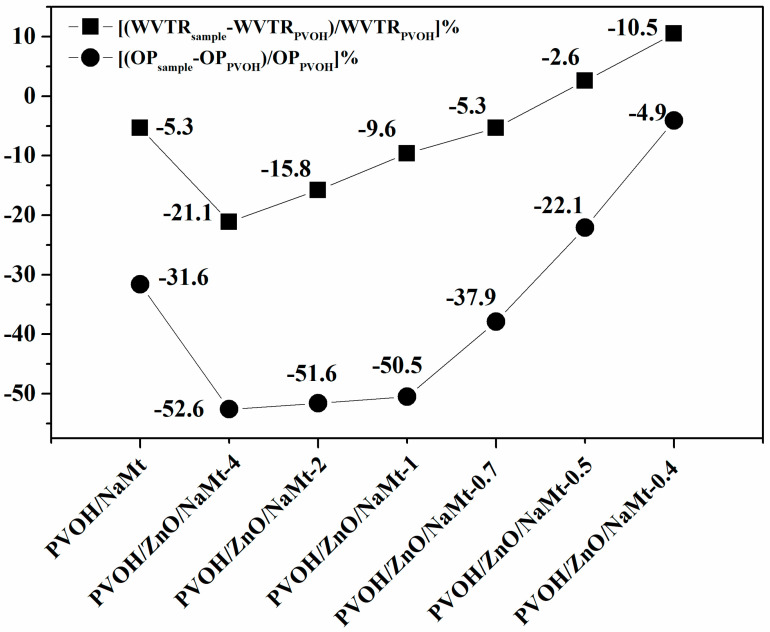
Comparison of the variation of % WVTR and % OP values of all the obtained PVOH-based nanocomposite films from the respective values of the pure PVOH film which was used as referenced material.

The PVOH polymer is known for its excellent oxygen barrier properties due to its strong intermolecular forces which originate from the hydroxyl groups in the repeating unit [40]. However, these hydroxyl groups make PVOH very hydrophilic and ultra-sensitive to moisture. WVTR values for PVOH films are rarely reported [40,41,42] because of their extreme weakness in moisture. Additionally, OTR values of PVOH films are rarely reported [40]. In this work, the PVOH films were prepared via a solution blending process and not via an extrusion melting process. Calculated WVTR and OP values of all the developed PVOH/ZnO/NaMt nanocomposites, as well as of the PVOH/NaMt nanocomposite and the pure PVOH, are listed in Table 4. The obtained WVTR values which are listed in Table 4 were measured with a handmade apparatus and thus could not be compared with other values reported in the literature. However, they were still useful for comparisons between the films of this work. In Figure 8, the % variation of the WVTR and OP values of each sample as compared to the WVTR and OP values of the pure PVOH sample is presented. This is a good indicator of water and oxygen barrier properties.

It is obvious from Table 3 and Figure 8 that the variation of WVTR and OP values follows the same trend. For PVOH/ZnO/NaMt-4, PVOH/ZnO/NaMt-2, and PVOH/ZnO/NaMt-1 nanocomposites obtained lower WVTR and OP values than for the PVOH/NaMt nanocomposite structure. The WVTR and OP values of PVOH/ZnO/NaMt-0.7, PVOH/ZnO/NaMt-0.5, and PVOH/ZnO/NaMt-0.4 nanocomposites samples were higher than WVTR and OP values of the PVOH/NaMt nanocomposite sample. In other words, PVOH/ZnO/NaMt nanocomposites with high ZnO nanorods content exhibited the highest oxygen and water barrier properties. The lowest WVTR and OP values were obtained for PVOH/ZnO/NaMt-4 and PVOH/ZnO/NaMt-2 nanocomposites samples. The PVOH/ZnO/NaMt-4 sample exhibited −21.1% and −52.6% lower WVTR and OP values, respectively, compared to the WVTR and OP values of the pure PVOH sample. Similarly, the PVOH/ZnO/NaMt-2 sample exhibited −15.8% and −51.6% lower WVTR and OP values, respectively, compared to the WVTR and OP values of pure PVOH sample. Moreover, similarly to the case of the tensile strength variation, both the WVTR and OP values variation of all nanocomposite samples were consistent with the variation of the d-spacing values which were recorded from XRD measurements. Thus, the PVOH/ZnO/NaMt-4, PVOH/ZnO/NaMt-2, and PVOH/ZnO/NaMt-1 nanocomposites which exhibited the highest d-spacing values and the strongest intercalated structure, also exhibited the highest barrier properties. On the other hand, the PVOH/ZnO/NaMt-0.5 and PVOH/ZnO/NaMt-0.4 samples, which exhibited the lowest d-spacing values and partially destroyed intercalated structure, also exhibited the lowest barrier properties. Although until now it was known that the addition of NaMt nanofiller to the PVOH matrix can increase only the water barrier properties [41], in this work it is reported that the addition of ZnO/NaMt nanohybrids can increase both water and oxygen barrier properties. The increase of oxygen barrier properties of PVOH/ZnO/NaMt nanocomposites should be attributed to the extra increase of the tortuosity of paths which are followed by the oxygen molecules because of the presence of ZnO nanorods on the silicate platelets. This fact results in a decrease of the effective diffusivity of oxygen through the films. On the other hand, the increase of the water barrier of the PVOH/ZnO/NaMt nanocomposites should be attributed to the water resistance properties of the ZnO nanorods [39]. These two results indicate that the followed preparation procedure is advantageous. This happened because of the ZnO nanorods growth on the external surface of NaMt layers.

### 3.6. UV–Vis Films Absorbance

**Figure 9:**
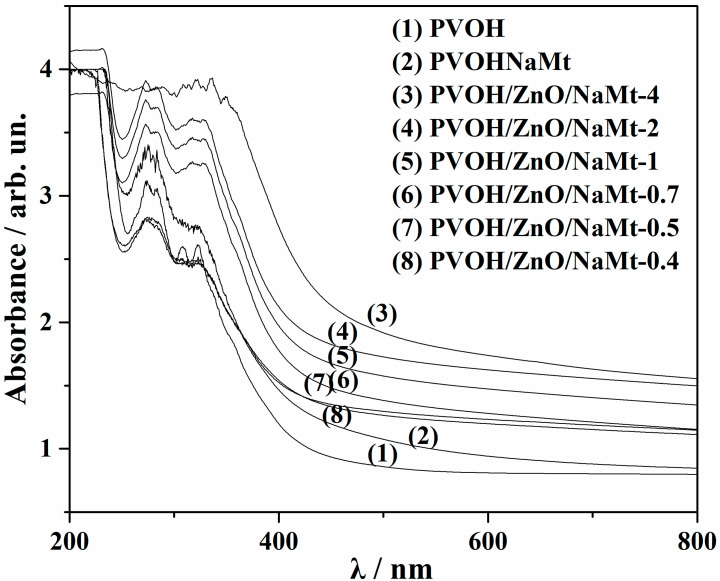
UV–vis absorbance plots of all the developed PVOH/ZnO/NaMt nanocomposite films as well as of the PVOH/NaMt nanocomposite and pure PVOH films.

The UV–vis absorbance measurements are a common and useful tool for the design of materials that are used for food product packaging [43,44,45]. As it is obvious from UV–vis absorbance plots in Figure 9, the pure POVH film (see dotted line spectra) absorbed a significant amount of UV–B light (280–315 nm) and a smaller amount of UV-A light (315−400 nm). The addition of ZnO/NaMt nanohybrids into the PVOH matrix enhances the visible light, UV-B, and UV-A irradiation absorption. As the ZnO content increases the UV-B and UV-A absorption increases also. For the PVOH/ZnO/NaMt-nanocomposite film with the highest ZnO nanorods, content a cut-off of UV irradiation is observed.

### 3.7. Antimicrobial Properties

**Figure 10:**
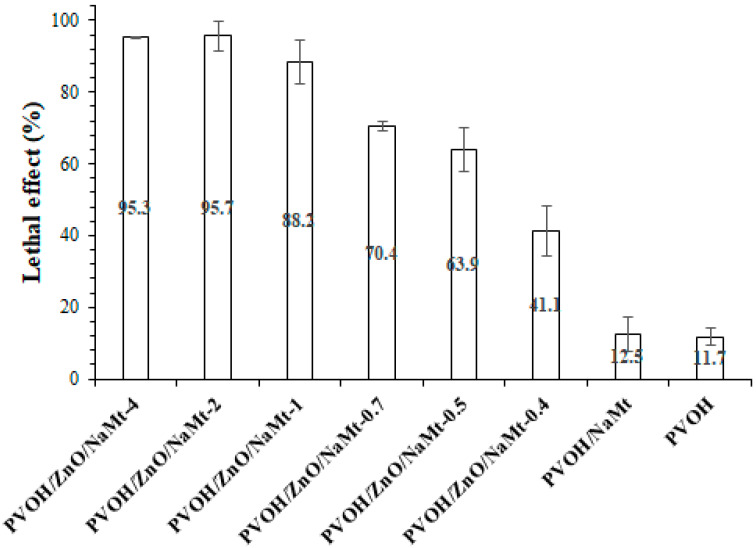
Lethal effect of all obtained PVOH/ZnO/NaMt nanocomposites films as well as of PVOH/NaMt nanocomposite and pure PVOH film against *Escherichia coli* after 12 h interaction at 25 °C. All measurements were in triplicate while the standard deviation (SD) is represented.

The effect of ZnO nanorods content on the antimicrobial activity of PVOH/ZnO/NaMt nanocomposite films against *E. coli* is presented in Figure 10. The bactericidal ability was studied through the lethal effect that was caused at ambient temperature (25 °C) after 12 h of interaction. Pure PVOH and PVOH/NaMt nanocomposite films had a light lethal effect of 12.5% and 11%, respectively. Pure NaMt bulk powder was tested and showed no significant antibacterial activity. Thus, the pure PVOH and the PVOH/NaMt materials exhibited identical antimicrobial activity (Figure 10) The lethal effect of PVOH/ZnO/NaMt-4 and PVOH/ZnO/NaMt-were almost 95%, whereas for PVOH/ZnO/NaMt-1. PVOH/ZnO/NaMt-0.7, PVOH/ZnO/NaMt-0.5, and PVOH/ZnO/NaMt-0.4 were 88, 70, 64, and 41, respectively. The antibacterial efficacy increased with increasing content (Table 2) of ZnO nanoparticles in the films. This is in agreement with a previously published report on the antibacterial properties of ZnO nanoparticles which showed that the antibacterial activity of ZnO nanoparticles increased with increasing powder concentration [46,47,48]. According to the results, it can be concluded that growing ZnO nanoparticles at PVOH/NaMt nanocomposite films are effective antibacterial agents against *E. coli*.

## 4. Conclusions

Concluding the above paragraphs, we could resume the following: (i) The NaMt and ZnO blending process, as it is described above, provides a nanostructured material with high antimicrobial activity. More specifically, the FTIR measurements confirm the ZnO/NaMt nanostructure and the PVOH/ZnO/NaMt ternary nanocomposite films. SEM images also confirm the coating of ZnO nanorods on the surface of the NaMt clay which permits the full intercalation of the clay by the PVOH polymeric matrix. (ii) Such nanostructured material exhibits enhanced enough antimicrobial properties compared to the PVOH/NaMt film because of the ZnO growth, as nanorods, on the external surface of the NaMt clay. The PVOH/ZnO/NaMt-4 material exhibits a lethal effect of almost 97% for *Escherichia coli* while the pure PVOH exhibits lethal effect around 10% for the same microbial and the NaMt exhibits almost 0%. (iii) Apart from that, compared to the PVOH/NaMt film, the PVOH/ZnO/NaMt nanocomposite material exhibits reduced tensile properties for low ZnO concentrations. This drawback was eliminated enough for higher ZnO concentrations. As it is confirmed by the tensile measurements, the mechanical properties of the PVOH/ZnO/NaMt-4 film are well improved compared to the mechanical properties of the PVOH/ZnO/NaMt-0.4 film. (iv) The oxygen permeation analyzer (OPA), the water sorption (WS), and the water vapor transition rate (WVTR) measurements, confirm the higher oxygen and water barrier properties of the PVOH/ZnO/NaMt-4 film compared to the oxygen and water barrier properties of the pure PVOH and the PVOH/NaMt materials. (v) Finally, the UV–vis absorbance measurements confirm the cut-off of the UV radiation by the PVOH/ZnO/NaMt film. According to the above-mentioned conclusions, the PVOH/ZnO/NaMt-4 film has great potential to be a promising active packaging film for food. A full migration and antimicrobial study for the evaluation of possible migration phenomena of such novel ZnO/NaMt material in food will be the subject of our next project.

## Figures and Tables

**Table 1:** Typical conditions and used ZN(CH3COO)_2_×2H_2_O amounts for the preparation of ZnO/NaMt nanohybrids.

A/A	Code Name	Zn(CH_3_COO)_2_×2H_2_O (g)	ZnO (g)	NaMt	Batch Total Volume (Water-mL)	Reflux (h)
1	ZnO	4.525	2.0	0.0	50	1
2	ZnO/NaMt-4	4.525	2.0	0.5	50	1
3	ZnO/NaMt-2	4.525	2.0	1.0	50	1
4	ZnO/NaMt-1	4.525	2.0	2.0	50	1
5	ZnO/NaMt-0.7	4.525	20	3.0	50	1
6	ZnO/NaMt-0.5	4.525	2.0	4.0	50	1
7	ZnO/NaMt-0.4	4.525	2.0	5.0	50	1

**Table 2:** Code names, used amounts of poly(vinyl)alcohol (PVOH) and ZnO/NaMt hybrids, % nominal contents in ZnO/NaMt nanohybrids, and extrusion processing conditions for all prepared active films.

Samples Code Names	PVOH (g)	ZnO/NaMt*(g)	%ZnO Content	%NaMt Content	Extrusion Process		
					T (°C)	Speed (rpm)	Time (min)
PVOH/ZnO/NaMt-4	4.85	0.15	2.4	0.6	190	100	10
PVOH/ZnO/NaMt-2	4.85	0.15	2.0	1.0	190	100	10
PVOH/ZnO/NaMt-1	4.85	0.15	1.5	1.5	190	100	10
PVOH/ZnO/NaMt-0.7	4.85	0.15	1.2	1.8	190	100	10
PVOH/ZnO/NaMt-0.5	4.85	0.15	1.0	2.0	190	100	10
PVOH/ZnO/NaMt-0.4	4.85	0.15	0.86	2.14	190	100	10
PVOH/NaMt	4.85	0.15	0.0	3.0	190	100	10

**Table 3:** Modulus of elasticity (E), tensile strength (σuts), and % elongation at break (%ε), of all tested PVOH/ZnO/NaMt nanocomposite films as well as of PVOH/NaMt nanocomposite and pure PVOH film.

Samples Code Names	Young’s Modulus-(E)	Tensile Strength-(σuts)	% Elongation at Break (%ε)
PVOH	2066.6 ± 146.5	6.7 ± 0.5	0.7 ± 0.1
PVOH/NaMt	2602.2 ± 158.8	12.5 ± 0.8	1.2 ± 0.2
PVOH/ZnO/NaMt-4	3154.3 ± 167.3	19.1 ± 0.7	1.0 ± 0.2
PVOH/ZnO/NaMt-2	2546.6 ± 158.5	12.7 ± 0.7	0.8 ± 0.2
PVOH/ZnO/NaMt-1	2328.8 ± 145.2	10.4 ± 0.6	0.8 ± 0.3
PVOH/ZnO/NaMt-0.7	2168.3 ± 163.2	6.3 ± 0.4	0.6 ± 0.1
PVOH/ZnO/NaMt-0.5	1978.2 ± 125.8	5.3 ± 0.4	0.4 ± 0.1
PVOH/ZnO/NaMt-0.4	936.5 ± 98.5	3.1 ± 0.3	0.3 ± 0.1

**Table 4:** % water sorption, water vapor transmission rate (WVTR), and oxygen permeability (OP) of all tested PVOH/ZnO/NaMt nanocomposite films as well as of PVOH/NaMt nanocomposite and pure PVOH films.

Samples Code Names	% Water Sorption	WVTR (g/m^2^/d)	OP (cm^3^.mm/m^2^/d)
PVOH	633.3 ± 5.5	30.1 ± 0.3	9.5 ± 0.4
PVOH/NaMt	626.3 ± 4.4	28.5 ± 0.4	6.5 ± 0.3
PVOH/ZnO/NaMt-4	66.5 ± 2.3	23.7 ± 0.3	4.5 ± 0.2
PVOH/ZnO/NaMt-2	155.9 ± 3.2	25.3 ± 0.3	4.6 ± 0.3
PVOH/ZnO/NaMt-1	165.2 ± 3.1	27.2 ± 0.2	4.7 ± 0.2
PVOH/ZnO/NaMt-0.7	220.2 ± 3.6	28.5 ± 0.2	5.9 ± 0.2
PVOH/ZnO/NaMt-0.5	290.5 ± 3.5	30.9 ± 0.3	7.4 ± 0.2
PVOH/ZnO/NaMt-0.4	310.3 ± 3.8	33.3 ± 0.3	9.1 ± 0.3

## References

[1] Bajpai V.K., Kamle M., Shukla S., Mahato D.K., Chandra P., Hwang S.K., Kumar P., Huh Y.S., Han Y.-K. (2018). Prospects of using nanotechnology for food preservation, safety, and security. J. Food Drug Anal..

[2] Giannakas A.E., Leontiou A.A. (2018). Montmorillonite composite materials and food packaging. Composites Materials for Food Packaging.

[3] Mohanty F., Swain S.K. (2017). Bionanocomposites for food packaging applications. Nanotechnol. Appl. Food FlavorStab. Nutr. Saf..

[4] Azizi-Lalabadi M., Ehsani A., Ghanbarzadeh B., Divband B. (2020). Polyvinyl alcohol/gelatin nanocomposite containing ZnO, TiO2 or ZnO/TiO2 nanoparticles doped on 4A zeolite: Microbial and sensory qualities of packaged white shrimp during refrigeration. Int. J. Food Microbiol..

[5] Liu X., Chen X., Ren J., Chang M., He B., Zhang C. (2019). Effects of nano-ZnO and nano-SiO2 particles on properties of PVA/xylan composite films. Int. J. Biol. Macromol..

[6] Mallakpour S., Madani M. (2012). Transparent and thermally stable improved poly (vinyl alcohol)/Cloisite Na+/ZnO hybrid nanocomposite films: Fabrication, morphology and surface properties. Prog. Org. Coat..

[7] Chen C., Xu Z., Ma Y., Liu J., Zhang Q., Tang Z., Fu K., Yang F., Xie J. (2018). Properties, vapour-phase antimicrobial and antioxidant activities of active poly(vinyl alcohol) packaging films incorporated with clove oil. Food Control..

[8] Jayakumar A., Heera K.V., Sumi T.S., Joseph M., Mathew S., Praveen G., Nair I.C., Radhakrishnan E.K. (2019). Starch-PVA composite films with zinc-oxide nanoparticles and phytochemicals as intelligent pH sensing wraps for food packaging application. Int. J. Biol. Macromol..

[9] Bumbudsanpharoke N., Ko S. (2019). Nanoclays in food and beverage packaging. J. Nanomater..

[10] Espitia P.J.P., Otoni C.G., Soares N.F.F., Barros-Velázquez J. (2016). Chapter 34—Zinc oxide nanoparticles for food packaging applications. Antimicrobial Food Packaging.

[11] Jia W., Dang S., Liu H., Zhang Z., Yu C., Liu X., Xu B. (2012). Evidence of the formation mechanism of ZnO in aqueous solution. Mater. Lett..

[12] Giannakas A., Patsaoura A., Barkoula N.-M., Ladavos A. (2017). A novel solution blending method for using olive oil and corn oil as plasticizers in chitosan based organoclay nanocomposites. Carbohydr. Polym..

[13] Giannakas A., Salmas C., Leontiou A., Tsimogiannis D., Oreopoulou A., Braouhli J. (2019). Novel LDPE/chitosan rosemary and melissa extract nanostructured active packaging films. Nanomaterials.

[14] Giannakas A., Xidas P., Triantafyllidis K.S., Katsoulidis A., Ladavos A. (2009). Preparation and characterization of polymer/organosilicate nanocomposites based on unmodified LDPE. J. Appl. Polym. Sci..

[15] Grigoriadi K., Giannakas A., Ladavos A.K., Barkoula N.-M. (2015). Interplay between processing and performance in chitosan-based clay nanocomposite films. Polym. Bull..

[16] Giannakas A., Giannakas A., Ladavos A. (2012). Preparation and characterization of polystyrene/organolaponite nanocomposites. Polym.-Plast. Technol. Eng..

[17] Giannakas A., Grigoriadi K., Leontiou A., Barkoula N.-M., Ladavos A. (2014). Preparation, characterization, mechanical and barrier properties investigation of chitosan–clay nanocomposites. Carbohydr. Polym..

[18] Giannakas A., Vlacha M., Salmas C., Leontiou A., Katapodis P., Stamatis H., Barkoula N.-M., Ladavos A. (2016). Preparation, characterization, mechanical, barrier and antimicrobial properties of chitosan/PVOH/clay nanocomposites. Carbohydr. Polym..

[19] Karageorgou D., Thomou E., Vourvou N.T., Lyra K.-M., Chalmpes N., Enotiadis A., Spyrou K., Katapodis P., Gournis D., Stamatis H. (2019). Antibacterial and algicidal effects of porous carbon cuboid nanoparticles. Acs Omega.

[20] Xu F., Yuan Z.-Y., Du G.-H., Ren T.-Z., Bouvy C., Halasa M., Su B.-L. (2006). Simple approach to highly oriented ZnO nanowire arrays: Large-scale growth, photoluminescence and photocatalytic properties. Nanotechnology.

[21] Hsu H.-C., Cheng C.-S., Chang C.-C., Yang S., Chang C.-S., Hsieh W.-F. (2005). Orientation-enhanced growth and optical properties of ZnO nanowires grown on porous silicon substrates. Nanotechnology.

[22] Khaorapapong N., Khumchoo N., Ogawa M. (2011). Preparation of zinc oxide–montmorillonite hybrids. Mater. Lett..

[23] Peng H., Liu X., Tang W., Ma R. (2017). Facile synthesis and characterization of ZnO nanoparticles grown on halloysite nanotubes for enhanced photocatalytic properties. Sci. Rep..

[24] Makarona E., Koutzagioti C., Salmas C., Ntalos G., Skoulikidou M.-C., Tsamis C. (2017). Enhancing wood resistance to humidity with nanostructured ZnO coatings. Nano-Struct. Nano-Objects.

[25] King S.L., Gardeniers J.G.E., Boyd I.W. (1996). Pulsed-laser deposited ZnO for device applications. Appl. Surf. Sci..

[26] Husain S., Alkhtaby L.A., Giorgetti E., Zoppi A., Muniz Miranda M. (2014). Effect of Mn doping on structural and optical properties of sol gel derived ZnO nanoparticles. J. Lumin..

[27] Akkari M., Aranda P., Ben Rhaiem H., Ben Haj Amara A., Ruiz-Hitzky E. (2016). ZnO/clay nanoarchitectures: Synthesis, characterization and evaluation as photocatalysts. Appl. Clay Sci..

[28] Xu H., Yu T., Liu J. (2014). Photo-degradation of Acid Yellow 11 in aqueous on nano-ZnO/Bentonite under ultraviolet and visible light irradiation. Mater. Lett..

[29] Wu L.M., Tong D.S., Zhao L.Z., Yu W.H., Zhou C.H., Wang H. (2014). Fourier transform infrared spectroscopy analysis for hydrothermal transformation of microcrystalline cellulose on montmorillonite. Appl. Clay Sci..

[30] Djomgoue P., Njopwouo D. (2013). FT-IR spectroscopy applied for surface clays characterization. J. Surf. Eng. Mater. Adv. Technol..

[31] Giannakas A., Tsagkalias I., Achilias D.S., Ladavos A. (2017). A novel method for the preparation of inorganic and organo-modified montmorillonite essential oil hybrids. Appl. Clay Sci..

[32] Anžlovar A., Orel Z.C., Kogej K., Zigon M. (2012). Polyol-mediated synthesis of Zinc Oxide nanorods and nanocomposites with Poly(methyl methacrylate). J. Nanomater..

[33] Soni B.H., Deshpande M.P., Bhatt S.V., Garg N., Chaki S.H. (2013). Studies on ZnO nanorods synthesized by hydrothermal method and their characterization. J. Nano Electron. Phys..

[34] Kandhol G., Wadhwa H., Chand S., Mahendia S., Kumar S. (2019). Study of dielectric relaxation behavior of composites of Poly (vinyl alchohol) (PVA) and Reduced graphene oxide (RGO). Vacuum.

[35] Ceran Ö.B., Şimşek B., Şara O.N. (2020). Preparation and characterization novel dioctyl terephthalate blended polyvinyl alcohol-composite films incorporated with the graphene oxide and silver nanoparticles. Polym. Test..

[36] Kashyap S., Pratihar S.K., Behera S.K. (2016). Strong and ductile graphene oxide reinforced PVA nanocomposites. J. Alloy. Compd..

[37] Luzi F., Di Michele A., Torre L., Puglia D. (2019). Active Role of ZnO Nanorods in thermomechanical and barrier performance of Poly(vinyl alcohol-co-ethylene) formulations for flexible packaging. Polymer.

[38] Abutalib M.M. (2019). Effect of zinc oxide nanorods on the structural, thermal, dielectric and electrical properties of polyvinyl alcohol/carboxymethyle cellulose composites. Phys. B Condens. Matter.

[39] Ennaceri H., Wang L., Erfurt D., Riedel W., Mangalgiri G., Khaldoun A., El Kenz A., Benyoussef A., Ennaoui A. (2016). Water-resistant surfaces using zinc oxide structured nanorod arrays with switchable wetting property. Surf. Coat. Technol..

[40] Lim M., Kwon H., Kim D., Seo J., Han H., Khan S.B. (2015). Highly-enhanced water resistant and oxygen barrier properties of cross-linked poly(vinyl alcohol) hybrid films for packaging applications. Prog. Org. Coat..

[41] Strawhecker K.E., Manias E. (2000). Structure and properties of Poly(vinyl alcohol)/Na+ montmorillonite nanocomposites. Chem. Mater..

[42] Hasimi A., Stavropoulou A., Papadokostaki K.G., Sanopoulou M. (2008). Transport of water in polyvinyl alcohol films: Effect of thermal treatment and chemical crosslinking. Eur. Polym. J..

[43] Hernández J.J., García-Gutiérrez M.C., Nogales A., Rueda D.R., Kwiatkowska M., Szymczyk A., Roslaniec Z., Concheso A., Guinea I., Ezquerra T.A. (2009). Influence of preparation procedure on the conductivity and transparency of SWCNT-polymer nanocomposites. Compos. Sci. Technol..

[44] Narayanan M., Loganathan S., Valapa R.B., Thomas S., Varghese T.O. (2017). UV protective poly(lactic acid)/rosin films for sustainable packaging. Int. J. Biol. Macromol..

[45] Babaei-Ghazvini A., Shahabi-Ghahfarrokhi I., Goudarzi V. (2018). Preparation of UV-protective starch/kefiran/ZnO nanocomposite as a packaging film: Characterization. Food Packag. Shelf Life.

[46] Amin K.M., Partila A.M., El-Rehim H.A.A., Deghiedy N.M. (2020). Antimicrobial ZnO nanoparticle–doped polyvinyl alcohol/pluronic blends as active food packaging films. Part. Part. Syst. Charact..

[47] Mirhosseini M., Firouzabadi F.B. (2012). Antibacterial activity of zinc oxide nanoparticle suspensions on food-borne pathogens. Int. J. Dairy Technol..

[48] Azam A., Ahmed A.S., Oves M., Khan M.S., Habib S.S., Memic A. (2012). Antimicrobial activity of metal oxide nanoparticles against Gram-positive and Gram-negative bacteria: A comparative study. Int. J. Nanomed..

